# Flushing of an intravenous catheter: a cause for unreliable laboratory results

**DOI:** 10.11613/BM.2019.031001

**Published:** 2019-08-05

**Authors:** Rutger C.C. Hengeveld, Maaike C. Gerards, Bianca E. Olofsen, Milan L. Ridderikhof, Victor F.H.A. Hakkenberg van Gaasbeek, Peter A. Leenhouts, Edmée C. van Dongen-Lases

**Affiliations:** 1 Department of Clinical Chemistry, Amsterdam UMC, University of Amsterdam, Amsterdam, Netherlands; 2 Department of Internal Medicine, Amsterdam UMC, University of Amsterdam, Amsterdam, Netherlands; 3 Department of Emergency Medicine, Amsterdam UMC, University of Amsterdam, Amsterdam, Netherlands

**Keywords:** preanalytical phase, phlebotomy, intravenous catheter

## Abstract

**Introduction:**

Phlebotomy is an error-prone process in which mistakes are difficult to reveal. This case report describes the effect on laboratory results originating from a blood sample collected in close proximity to an intravenous catheter.

**Materials and methods:**

A 69-year-old male patient was referred to the Emergency department where pneumonia was suspected. Phlebotomy was performed to collect blood samples to assess electrolytes, renal function, liver function, infection and haematological parameters.

**Results:**

The laboratory analysis showed reduced potassium and calcium concentrations. To prevent life-threatening cardiac failure the clinician decided to correct those electrolytes. Remarkably, the electrocardiogram showed no abnormalities corresponding to hypokalaemia and hypocalcaemia. This observation, in combination with an overall increase in laboratory parameters with the exception of sodium and chloride, led to the suspicion of a preanalytical error. Retrospectively, an intravenous catheter was inserted in close proximity of the puncture place but no continuous infusion was started prior to phlebotomy. However, the intravenous catheter was flushed with sodium chloride. Since potential other causes were excluded, the flushing of the intravenous catheter with sodium chloride prior to phlebotomy was the most probable cause for the deviating laboratory results and subsequently for the unnecessary potassium and calcium suppletion.

**Conclusion:**

This case underlines the importance of caution in the interpretation of laboratory results obtained from specimens that are collected in the proximity of an intravenous catheter, even in the absence of continuous infusion.

## Introduction

The preanalytical phase includes procedures concerning sample collection prior to laboratory analysis such as patient identification, phlebotomy and transport of specimens to the laboratory (*1*). Sample haemolysis due to usage of inappropriate needles, under filling of collection tubes, usage of wrong collection tubes and partial coagulation are common examples that potentially influence clinical laboratory outcomes (*2*). In particular, phlebotomy is an error-prone process that should be performed by well-trained certificated phlebotomists (*3*, *4*). Sampling errors are the major cause for hardly traceable unreliable laboratory results which can easily lead to misdiagnosis and wrong treatment of patients. In such cases the interferences could be subtle and therefore not always noticed by laboratory technicians, clinical chemists and clinicians. Despite experienced laboratory experts being aware of such pitfalls, it is important to gain insight in cases in which erroneous phlebotomy causes unreliable laboratory results. This case report describes the risks and consequences of performing a venipuncture in close proximity to an intravenous catheter.

## Case report and laboratory analyses

### Patient medical history

The described patient provided written informed consent. A 69-year-old male patient was referred to the Emergency department (ED) of our university hospital. The past medical history mentions pleuropneumonia and tuberculous meningitis 21 years prior to presentation. A follicular lymphoma was diagnosed 8 years later for which he was treated with chemo-immunotherapy and allogenic stem cell transplantation. Subsequent immune suppression therapy to minimize graft *versus* host disease after allogenic stem cell transplantation was complicated by an episode of meningitis and pneumonia. Because of this past medical history he was checked at the Haematology Department on a regular basis, during which laboratory analysis was performed to monitor renal and liver function and haematological parameters. At the most recent visit three months prior to presentation no clinically relevant abnormal laboratory results were obtained (Table 1).

**Table 1:** Laboratory results prior and during patient presentation at the Emergency department.

	**Results**			
**Parameter, unit**	**3 months prior presentation**	**Admission at ED**	**Post potassium and calcium suppletion**	**Reference range**
Sodium, mmol/L	139	143	135	135-145
Potassium, mmol/L	4.8	2.5	5.6	3.5-4.5
Calcium, mmol/L	ND	1.32	2.26	2.20-2.60
Phosphate, mmol/L	ND	0.31	1.02	0.81-1.45
Chloride, mmol/L	ND	119.5	102	98-107
Magnesium, mmol/L	ND	0.46	0.83	0.75-1.03
Urea, mmol/L	ND	6.6	9.6	2.9-8.2
Creatinine, μmol/L	ND	67	105	75-110
AST, U/L	ND	147	230	0-40
ALT, U/L	23	149	247	0-45
ALP, U/L	80	110	188	40-120
GGT, U/L	37	83	135	0-60
Albumin, g/L	ND	21.6	35	35-50
CRP, mg/L	ND	59.1	111.5	0-5
Transferrin, g/L	ND	1.01	ND	2-3.6
Vitamin B12, pmol/L	ND	219	ND	150-700
Ferritin, μg/L	ND	422	ND	25-300
Haemoglobin, g/L	134	90	123	137-169
Leukocytes, x10^9^/L	12.5	8.67	11.4	4-10.5
Thrombocytes, x10^9^/L	268	146	218	150-400
ND - not determined.				

### Emergency department presentation

**Figure 1:**
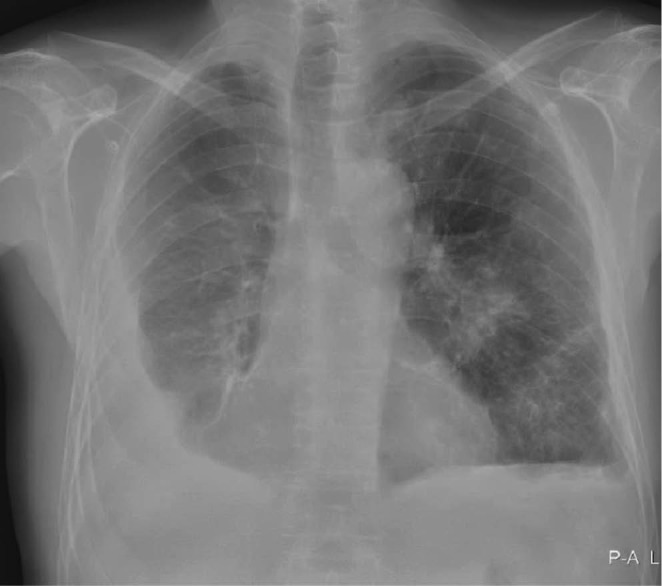
Chest X-Ray after patient presentation at the Emergency department.

The reason for patient presentation at the ED was suspicion of pneumonia with complaints of dyspnoea, without signs of coughing. Moreover, he experienced weakness in his legs since one week. During physical examination, the patient had a body temperature of 39.6 degrees Celsius and an oxygen saturation of 96% during three litres oxygen supplementation *per* minute, but was not in acute respiratory distress. Percussion dullness was noticed at the lower right thorax during pulmonary examination. The chest X-ray was markedly abnormal with flattening of the left hemi-diaphragm and pleural effusion and atelectasis in the right hemithorax was observed, but this was in accordance with previous observed pleurodiaphragmatic adhesions (Figure 1). The abnormalities at the left were slightly increased compared to a previous observation.

### Laboratory analysis and patient’s treatment

**Figure 2:**
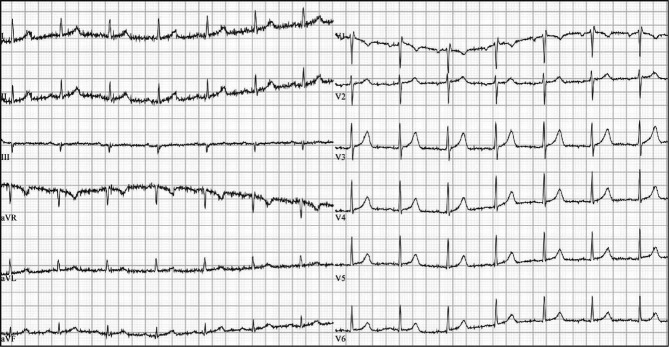
ECG at the Emergency department presentation: a sinus rhythm of 75 beats *per* minute with an intermediate heart axis without any conduction abnormalities. No ST elevation or depression and normal T-wave morphology.

To support the diagnostics, the phlebotomist collected blood samples and sent these to the laboratory for haematological and chemical analysis to assess electrolytes, renal function, liver function, infection and haematological parameters. The haematological parameters were measured on an automated XN-9000 haematology analyzer (Sysmex, Norderstedt, Germany). A Cobas C-8000 automated modular analyzer (Roche Diagnostics, Indianapolis, USA) was used to perform chemical analysis. No technical errors during laboratory analysis were reported. Strikingly reduced concentrations of potassium were observed (Table 1). Therefore, the clinician decided to supplement the patient with 40 mmol potassium orally to prevent serious rhythm abnormalities. In addition, 2.25 mmol calcium gluconate was supplemented intravenously based on the low concentration of calcium analysed from the same blood sample. To investigate whether the hypokalaemia and hypocalcaemia caused cardiac conduction disturbances an electrocardiogram (ECG) was performed. However, no abnormalities consistent with low potassium or low calcium were observed (Figure 2).

## What happened?

**Figure 3:**
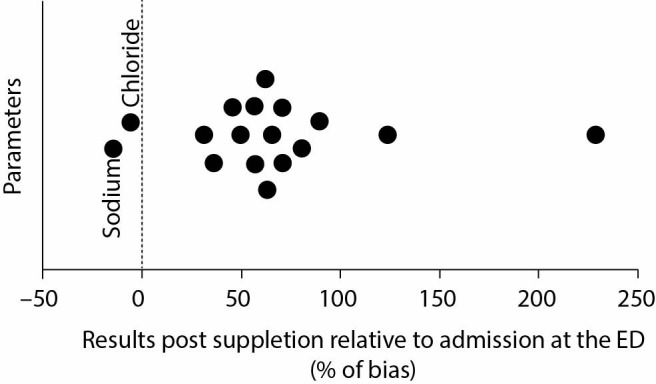
Laboratory results post electrolyte suppletion compared to admission at the Emergency department. The differences in laboratory outcomes post potassium and calcium suppletion relative to admission at the Emergency department were calculated and shown as percent of bias. Sodium and chloride are pointed out.

The results of the ECG recording combined with the elevated potassium and calcium concentrations measured from the sample post-suppletion (Table 1), raised the suspicion of a preanalytical error. To investigate whether an incorrect preanalytical proceeding was the cause of the deviating laboratory outcomes, the test results before and after potassium and calcium suppletion were compared. Strikingly, after suppletion, an increase in electrolytes, proteins, leukocytes and thrombocytes were observed in specimens collected post electrolyte suppletion (Table 1). Since the laboratory parameters at admission at the ED showed an overall reduction, the clinician suspected that an incorrect phlebotomy could be the underlying cause of the deviating laboratory results. Therefore, the phlebotomist who performed the specimen collection was asked for potential factors during phlebotomy that could have affected the laboratory outcomes. The median cubital vein was selected for venipuncture. Just before venipuncture, an intravenous catheter was inserted (exact time interval unknown) in close proximity distal of the puncture place. No continuous infusion was started, but the catheter was flushed with 10 mL sodium chloride. According to the phlebotomist other potential causes could be excluded. In contrast to other laboratory parameters, sodium and chloride were decreased post electrolyte suppletion, which was indicative for sodium chloride contamination (Table 1 and Figure 3). It was concluded that admixture of sodium chloride by flushing of the intravenous catheter prior to phlebotomy was the underlying cause for the deviating laboratory results and subsequently unnecessary suppletion of electrolytes.

The suppletion of potassium and calcium was ceased immediately and fortunately the patient suffered no adverse effects from the wrong treatment.

## Discussion

Phlebotomy should be performed by well-trained personnel according to well-established valid procedures (*5*-*7*). A wide variety of requirements for flawless phlebotomy must be taken into account. These include the use of suitable needles and sample collection tubes, proper tourniquet use, selecting a proper vein for needle insertion, a proper mixture with additives and correct controlled storage and transport of blood samples (*8*). The fact that phlebotomy should be avoided in close proximity of an inserted intravenous catheter has been described previously (*9*). The Dutch national guideline on phlebotomy dictates that an active intravenous catheter should be stopped during at least two minutes prior to venipuncture (*10*). It is recommended that this procedure should be reported and taken into account during interpretation of laboratory results, in case of unexpected aberrant laboratory results. This case report underlines the importance of accuracy of the phlebotomy process and of the analysis of the laboratory results. We showed that flushing of an intravenous catheter with 10 mL sodium chloride is sufficient to generate aberrant laboratory results. This had led to unjustified and excessive potassium and calcium suppletion which could have been life threatening. Similar aberrant results of sodium, potassium and chloride were observed in a previous case in which normal saline was infused continuously prior phlebotomy (*9*). Due to risk of generating unreliable laboratory results after flushing of an intravenous catheter it is of great importance that the phlebotomist proceeds according to an established procedure. Therefore, we recommend a stopping interval of at least two minutes not only in case of a continuous infusion but also between flushing the infusion and the procedure of phlebotomy. It should be kept in mind that the time interval of two minutes is an expert opinion/level D evidence and not based on experimental evidence. Phlebotomy is, in spite of adhering to strict procedures as well as thorough training of phlebotomists, a vulnerable process. Potential mistakes can easily go unnoticed. Therefore, knowledge of preanalytical pitfalls and alertness of clinicians and authorizers of laboratory results are essential to detect unnoticed errors in the preanalytical phase. It should be a duty of the laboratory professionals to inform the clinicians and nurses on a regular basis to aware them of the potential risks in the preanalytical phase. In the current case report, the abnormal laboratory results and the ECG were the trigger for the clinician to suspect contamination with sodium chloride from the inserted intravenous catheter. Decreased potassium, calcium, phosphate and magnesium combined with normal sodium and chloride concentrations must be a trigger for the laboratory personal to inform the clinician and to request a new blood sample.

Therefore, in addition to accuracy during phlebotomy, assessing the entire dataset of laboratory results instead of focusing on the aberrant results are both essential to track down phlebotomy related preanalytical causes of unreliable laboratory outcomes. Communication between treating medical staff and the laboratory department is essential to track errors in the preanalytical phase.

## What YOU should/can do in your laboratory to prevent such errors

Introduction of a stopping interval of at least two minutes not only in case of a continuous infusion but also between flushing the infusion and the procedure of phlebotomy.

Regularly inform the clinicians and nurses how to track down preanalytical causes of unreliable laboratory outcomes.
